# Anticipating different grips reduces bimanual end-state comfort: A tradeoff between goal-related and means-related planning processes

**DOI:** 10.1371/journal.pone.0190586

**Published:** 2018-01-08

**Authors:** Christian Seegelke, Matthias Weigelt

**Affiliations:** 1 Biopsychology and Cognitive Neuroscience, Faculty of Psychology and Sports Science, Bielefeld University, Bielefeld, Germany; 2 Neurocognition and Action Research Group, Faculty of Psychology and Sports Science, Bielefeld University, Bielefeld, Germany; 3 Center of Excellence Cognitive Interaction Technology (CITEC), Bielefeld, Germany; 4 Psychology and Movement Science, Department of Sport and Health Sciences, Faculty of Science, Paderborn University, Paderborn, Germany; University of Exeter, UNITED KINGDOM

## Abstract

The present study explored the sensitivity towards bimanual end-state comfort in a task that required anticipating different final grips. Participants simultaneously reached and grasped two objects with either a whole-hand grip (WHG) or a precision grip (PG), and placed them at two target locations by transporting them either over or under an obstacle. The transport path was varied such that it could be either congruent (i.e., both objects over or under) or incongruent (i.e., one object over and the other object under). In the congruent conditions, participants satisfied bimanual end-state comfort (and identical initial grips) on the majority of trials. That is, participants adopted a PG for either hand when the objects were transported over the obstacle and a WHG for either hand when the objects were transported under the obstacle. In contrast, in the incongruent conditions, bimanual end-state comfort was significantly reduced, indicating the presence of intermanual inference. The results indicate that goal-related planning constraints (i.e., bimanual end-state comfort) do not strictly take precedence over means-related constraints (i.e., identical initial grips) if this requires anticipating different final grips. Thus, bimanual end-state comfort *per se* does not provide a predominant constraint in action selection, by which sensorimotor interference can be reduced. In line with the proposal that bimanual grip planning relies on a flexible constraint hierarchy, a simple formal model that considers bimanual grip posture planning as a tradeoff between goal-related and means-related planning processes can explain our results reasonably well.

## Introduction

A core challenge for any theory of action selection is to determine how actions are selected from an almost infinite number of alternatives to solve a particular movement task (cf. [1]). When reaching and grasping a coffee mug, one must determine, for example, the limb to reach with, where to grasp the mug, how fast to reach towards the mug, and so on. One approach that has been pursued to tackle this problem is to examine factors–hereafter referred to as constraints—that consistently influence action selection. Constraints make certain actions more likely to be selected, hence, they limit the range of possible action alternatives [2]. In the case of the coffee mug example, most people would certainly prefer to grasp the mug with their dominant hand, whereas they would presumably put less emphasis on the speed at which to approach the object. This highlights another aspect: That is, not all constraints are equally important, but constraints may be weighted according to their importance, constituting a ranking of constraints, or what has been called a constraint hierarchy [3]. Consequently, researchers are not only interested to identify action selection constraints, but also to examine which constraints are more important and receive more weight.

This approach has been frequently utilized and has provided valuable insights into action selection processes, specifically in the context of object manipulation tasks. Here, it has been demonstrated that participants grasp the same object differently, depending on what they intend to do with the object (see [4] for a review). In an influential study [5], participants reached out and grasped a horizontal bar to place either its left or right end down onto a target. The authors found that participants always selected the initial grip posture (overhand vs. underhand grip) in such a way that it afforded a comfortable final posture. This so called end-state comfort (ESC) effect has been confirmed in a number of different studies (e.g., [6–11]), and hence is considered a prominent action selection constraint during unimanual object manipulation. More generally, this phenomenon has been taken to support the notion that participants select initial grip postures in anticipation of future body states, and that actions are represented in terms of goal states [3].

More recently, researchers have designed experiments to contrast two (or more) action selection constraints to assess their relative weighting. In order to determine a ranking of constraints, the typical experimental procedure involves creating situations in which not all constraints can be satisfied at the same time. This way, it can be decided which of the different constraints are preferred over other constraints. Following this procedure, it has been shown, for example, that people typically assign more weight to end-state comfort than to manipulate an object with their dominant hand [12] (see also [13] for a similar conclusion using a different methodology).

Another interesting scenario which researchers have capitalized on to pit different action selection constraints against each other is bimanual coordination. One intriguing query that has been frequently addressed in the past years, and which also constitutes the focus of the present study, is whether people would also plan for end-state comfort when they have to manipulate two objects simultaneously [14–17]. This question was theoretically interesting, because previous research on bimanual coordination has demonstrated that utilizing different means (i.e., different pattern of muscle activation for each arm) can considerably affect performance. Such means-related influences are, for example, expressed in the strong tendency for the two hands to stay spatially and temporally coupled (i.e., bimanual symmetry; [18,19]). Furthermore, the simultaneous preparation of incongruent bimanual movements can result in a substantial increase in response times, as compared to the preparation of congruent bimanual movements (e.g., [20,21]). However, these sensorimotor intermanual inference effects seem to strongly depend on how the action goals are represented and can be virtually abolished when the actions are directly specified or conceptualized to focus on a simplified (sensory) goal [22,23]. For example, in one study [24], participants grasped two objects and turned them into either parallel (congruent) or opposite (incongruent) goal orientations, using either mirror-symmetrical or mirror-asymmetric movements. The authors found that performance was strongly determined by the intended goal congruency of the two objects, but virtually unaffected by the specific movements performed. In more general terms, they concluded that bimanual performance is not constrained by the inherent properties of the motor system, but rather by the intended goal states.

Weigelt and colleagues [17] were amongst the first to examine the interaction between end-state comfort and initial means (i.e., identical initial grips), using a bimanual version of the bar-transport task [5]. In the non-critical conditions of this study, the two constraints (i.e., end-state comfort and identical initial grips) coincided. However, in the critical conditions, the two constraints were put in conflict with each other, such that participants could either adopt different initial grips to satisfy end-state comfort, or could adopt identical initial grips at the cost of not satisfying end-state comfort for both hands. The authors found that participants satisfied end-state comfort for both hands, even if they had to adopt different initial grip postures. These findings further reinforced the fundamental role of intended goal states in selecting suitable movements and supported the view that means (i.e., initial grips) are prepared relative to desired ends (i.e., comfortable end-postures). Accordingly, Weigelt and colleagues [17] argued that the anticipation of a common action goal (i.e., bimanual end-state comfort) determines the production of appropriate motor actions with little interference between the hands.

It is important to note, that in the study of Weigelt and colleagues [17], bimanual end-state comfort was achieved by anticipating and adopting identical final grips. To the best of our knowledge, no study has explored whether goal-related planning constraints (i.e., end-state comfort) will also take precedence over means-related constraints (i.e., identical initial grips) if this requires anticipating different final postures of the two hands.

To approach this question, we employed a paradigm in which participants simultaneously reached and grasped two objects from two start locations and placed them at two target locations, by transporting them either over or under an obstacle. The objects were designed in such a way that they could be grasped either at their lower parts with a power grip–hereafter referred to as whole-hand grip (WHG)–or at their upper parts with a precision grip (PG; [25]). A WHG was considered to satisfy end-state comfort when the object had to be moved under the obstacle and a PG to satisfy end-state comfort when the object was moved over the obstacle. As a baseline and to verify this hypothesis, data from unimanual trials were obtained in which both the hand (left, right) and the transport path (over, under) were varied. For the bimanual task, the transport path for the two objects was varied in such a way that it could be either congruent [i.e., both objects over (OO) or under (UU) the obstacle] or incongruent [i.e., left object over and right object under (OU) or left object under and right object over (UO) the obstacle]. Thus, in the congruent trials, bimanual end-state comfort coincided with initial means (i.e., the selection of identical grips would also satisfy end-state comfort for both hands). In contrast, in the incongruent trials, participants could either adopt different grips to satisfy bimanual end-state comfort or adopt identical initial grips, but not satisfy bimanual end-state comfort.

If goal-related planning constraints (i.e., end-state comfort) take precedence over means-related constraints (i.e., identical initial grips), we expected to observe high and similar bimanual end-state comfort values during bimanual congruent and bimanual incongruent conditions, indicating that WHGs and PGs can be generated independently (End-state dominance hypothesis). In contrast, if means-related constraints dominate the selection of appropriate grips, we expected to observe bimanual end-state comfort values to be close to zero during bimanual incongruent conditions, and thus considerably lower compared to the bimanual congruent conditions, indicating planning interference between the concurrent selection of WHGs and PGs (Means dominance hypothesis).

Furthermore, the End-state dominance hypothesis predicts similar reaction times (RTs) and movement times (MTs) between bimanual congruent and bimanual incongruent conditions, whereas the Means dominance hypothesis predicts slower RTs and MTs for bimanual incongruent compared to bimanual congruent conditions.

## Method

### Ethics statement

The experiment was conducted in accordance with the ethical guidelines stated within the declaration of Helsinki and was approved by the ethics committee at Bielefeld University. All participants gave their informed written consent to participate in the study.

### Participants

40 right-handed individuals (mean handedness score = 98.25 SD = 9.19; [26]) from Bielefeld University (mean age = 25.43 years, SD = 3.55, 14 female, 26 male) participated in exchange for 5 € or course credit. All participants had normal or corrected to normal vision and were physically and neurologically healthy.

### Apparatus

**Fig 1:**
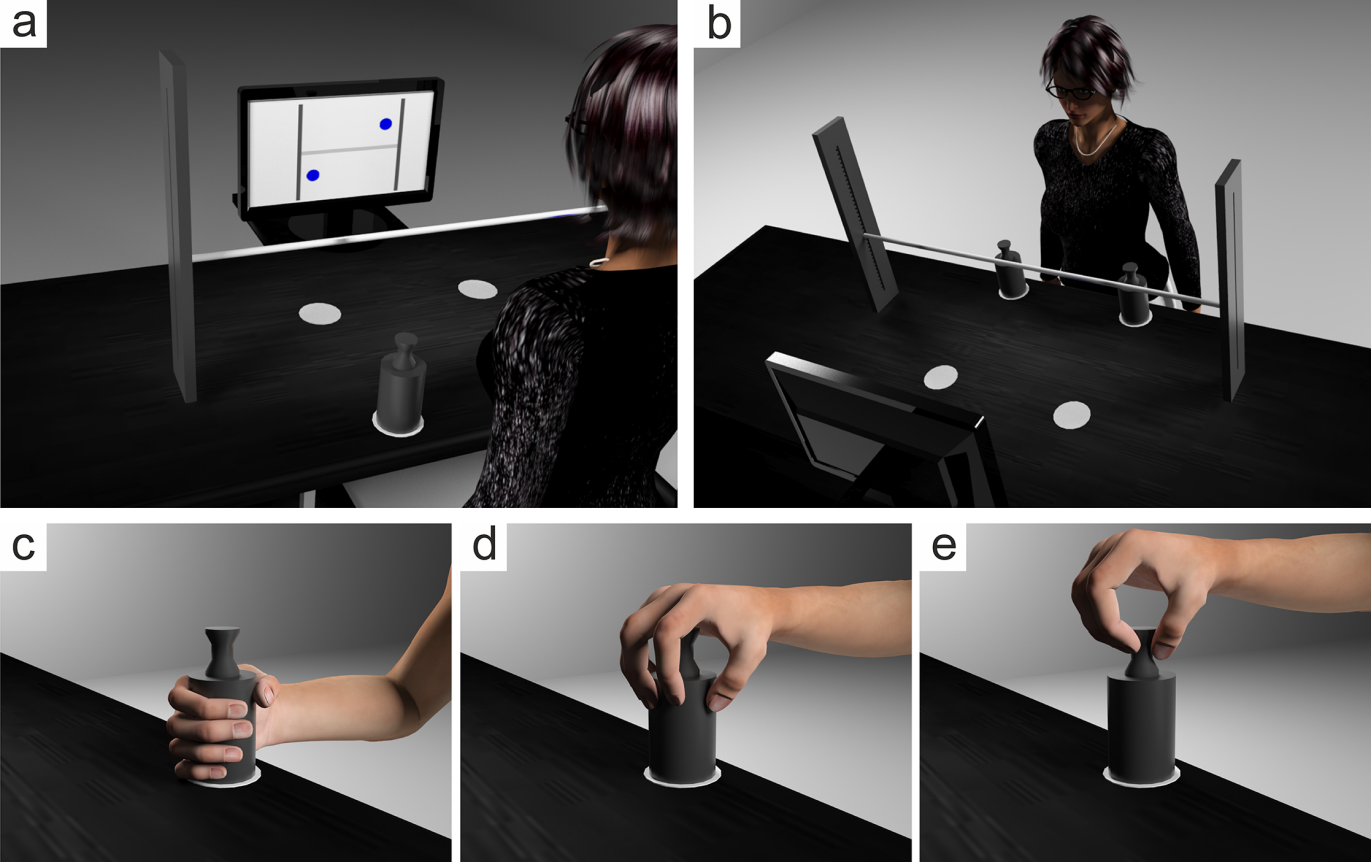
Experimental setup and observed grip types adopted by the participants. (a) Front view of the experimental setup, displaying a stimulus indicating the required transport paths for a bimanual trial in which the left object had to be moved under the obstacle and the right object had to be moved over the obstacle (UO condition). (b) Top view of the experimental setup. (c) Whole-hand grip (WHG). (d) 5-finger precision grip (PG-5). (e) 3-finger precision grip (PG-3). The experimental setup and the participants shown in the figure were created using the 3D graphics software 3Ds Max (Autodesk) and Poser (Smith Micro Software).

The experimental setup was positioned on a shelf of adjustable height (200 cm x 60 cm, Fig 1). A horizontal bar (80 cm in length, 1 cm in diameter), braced by two legs, which held the bar 20 cm above the shelf, served as the obstacle. It was positioned 20 cm from the front edge of the shelf. White paper circles (7 cm in diameter) were taped flat to the surface of the shelf, indicating the start and target positions. The start and target positions were spaced 30 cm apart and located 15 cm in front or behind the obstacle, respectively. The objects were two grey PVC cylinders consisting of a lower part (6 cm in diameter, 9 cm in height) and an upper part (3 in diameter, 4 cm in height).

Visual stimuli were presented on a 43 cm computer display (SyncMater 943T, Samsung) that was placed 40 cm behind the obstacle. The stimuli consisted of a visual representation of the setup and displayed the required transport path (i.e., over/ under the obstacle; Fig 1). Stimulus presentation was controlled via Presentation® (Neurobehavioral Systems).

An optical motion capture system (Vicon Motion Systems, Oxford, UK) was used to record kinematic data. It consisted of 10 Bonita cameras with a temporal and spatial resolution of 200 Hz and 1mm, respectively. Three retro-reflective markers (14 mm in diameter) were placed on the distal end of the third metacarpal (MCP), the styloid process of the radius (WRT), and the styloid process of the ulna (WRP) of each hand. Each trial was further recorded using a Basler Pilot DV camera (Basler AG, Ahrensburg, Germany), that was placed above the apparatus, providing a bird’s eye view of the apparatus and the participant. The video camera was used to record grip selection. The Vicon motion capture system was synchronized with both the digital video camera and the stimulus presentation system.

### Tasks and procedure

After reading and filling out the written consent and handedness inventory forms, participants’ hip height was measured, and the shelf was adjusted to hip height. Participants stood in front of the experimental setup so that their body midline coincided with the center of the obstacle at a distance so that they could comfortable reach the target positions.

They were tested in two task versions: In the unimanual task, participants reached and grasped only one object with either the left or the right hand from the start position and placed it to the target position by transporting it either over or under the obstacle (as indicated by the stimulus). In the bimanual task, participants reached and grasped for the two objects simultaneously from the start positions and placed them to the target positions by transporting them either both over (OO), both under (UU), or one over and the other under the obstacle (OU, UO), depending on task condition. For both tasks, participants always grasped the object located at the left start position with the left hand and placed it at the left target position, and vice versa for the object located at the right target position.

At the start of each trial, participants closed their eyes and the experimenter placed the objects at the start positions. The participants then opened their eyes and the message “Prepare for the next trial!” (in German) was displayed, which prompted participants to place their hands by their sides. A fixation cross was then presented for 500 ms, and after a random time interval (500–1500 ms), the stimulus was displayed for 500 ms. The appearance of the stimulus display served as the imperative signal. Participants grasped the object(s) from the start position(s) and placed it (them) to the target position(s) by transporting it (them) via the required transport path (over or under the obstacle), as indicated by the stimulus. At the end of each trial, participants brought their hand(s) back to the side of the body and waited for the next trial to begin. Participants were instructed to perform the tasks as quickly as possible while placing the object(s) accurately at the target position(s) and without hitting the obstacle during object transport.

For the unimanual task, there were four different conditions comprised of the factors hand (left, right) and transport path (over, under). For the bimanual tasks, the transport paths were either identical (OO, UU) or different (OU, UO), resulting in another four different conditions. The order of the task versions (unimanual vs. bimanual task) was blocked and counterbalanced across participants. Within each task, each condition was repeated 5 times in a randomized order, resulting in a total of 40 trials (20 unimanual, 20 bimanual).

### Data processing and analysis

The 3D coordinates of the retro-reflective markers were reconstructed and labeled. Missing data (gaps of less than 10 frames) were interpolated using a cubic spline, with larger gaps interpolated using the pattern fill algorithm within Vicon Nexus. The data were filtered using a Woltring filter [27] with a predicted mean square error of 5 mm^2^ (Vicon Nexus 1.8.2). Kinematic variables were calculated using a custom written MatLab script (The MathWorks, Version R2010a). The wrist joint center (WJC) of each hand was calculated as the midpoint between WRT and WRP.

For each trial, the reach-to-grasp phase was defined as the time period between when the hand (WJC) left the body to the time the hand (WJC) contacted the object. Reach-to-grasp onset was determined as the time of the sample in which the resultant velocity of the hand (WJC) exceeded 5% of reach-to-grasp peak velocity. Reach-to-grasp offset was determined as the time of the sample in which the resultant velocity dropped and stayed below 5% of reach-to-grasp peak velocity. Reaction time (RT) was defined as the time period between stimulus onset and reach-to-grasp onset. Movement time (MT) was defined as the time period between reach-to-grasp onset and reach-to-grasp offset.

Trials in which participants hit the obstacle (0.6%), used the wrong transport path (0.4%), used the wrong hand (0.1%), or in which they performed the bimanual task sequentially (0.1%) were excluded from analyses. In addition, we excluded trials in which RT and/or MT fell outside three times the interquartile range for each condition (1.8%). This procedure yielded that for two participants there were less than three valid observation in one or more experimental conditions. Consequently, these participants were removed from the analyses.

## Results

### Grip selection

First inspection of the video data revealed that participants adopted three different grip types (Fig 1). Participants either grasped the object from the side at its lower part using a WHG (Fig 1C), from the top at its lower part using a 5-finger PG (PG-5, Fig 1D), or from the top at its upper part using a 3-finger PG (PG-3, Fig 1E). Overall, participants selected a WHG in 54.3%, a PG-5 in 29.0%, and a PG-3 in 17.7% of the trials (see Table 1 for a detailed distribution of the three grip types for unimanual and bimanual trials as a function of hand and transport path). For further analyses, data for the PG-3 and PG-5 were pooled to form a single PG category. A WHG was considered to satisfy end-state comfort when the object had to be moved under the obstacle and a PG to satisfy end-state comfort when the object was moved over the obstacle.

**Table 1:** Distribution of the three observed grip types (in percent) as a function of task, hand, and transport path.

Unimanual					Bimanual congruent (OO and UU)					Bimanual incongruent (OU and UO)				
		Grip type					Grip type					Grip type		
		WHG	PG-5	PG-3			WHG	PG-5	PG-3			WHG	PG-5	PG-3
**Over**					**Over**					**Over**				
	**Left hand**	16.6	53.5	29.9		**Left hand**	19.9	54.8	25.3		**Left hand**	21.3	56.8	21.9
	**Right hand**	12.2	54.5	33.3		**Right hand**	18.8	57.5	23.7		**Right hand**	22.5	57.1	20.3
**Under**					**Under**					**Under**				
	**Left hand**	89.4	0.5	10.1		**Left hand**	91.4	0.5	8.1		**Left hand**	84.6	3.8	11.5
	**Right hand**	89.8	0.0	10.2		**Right hand**	90.9	1.1	8.1		**Right hand**	82.0	8.2	9.8

To verify this consideration, we obtained comfort ratings from an independent sample (n = 15, mean age = 22.40 years, SD = 2.53, 10 women, 5 men). The experimental setup was identical to that used in the main experiment. At the start of each trial, participants were instructed with which hand (left, right) and grip (WHG, PG-5, PG-3) to grasp the object. After grasping the object from the start position, participants provided a rating of the initial grip comfort on a scale ranging from 1 (very uncomfortable) to 5 (very comfortable). Participants were then instructed about the movement path (over, under) and after having placed the object at the target position via the instructed path, they provided a second rating of comfort with respect to the final posture. Initial and final comfort was analyzed using separate repeated measures ANOVAs with the within-subject factors hand (left, right), movement path (over, under), and grip (WHG, PG-5. PG-3). For initial comfort, there was only a significant main effect of grip, F(2,28) = 8.86, p = 0.001. Post hoc tests indicated that WHGs (mean comforting rating = 4.9) were perceived as more comfortable than PG-5 (mean comfort rating = 4.2, p = 0.002). Of major importance, for final comfort, the grip × path interaction was significant, F(2,28) = 73.75, p < 0.001. Simple effect analyses indicated that PG-3 and PG-5 were perceived as more comfortable than WHGs when the object was moved over the obstacle (PG-3 = 4.5, PG-5 = 3,8, WHG = 2.1; both p < 0.001). In contrast, when the object was moved under the obstacle, WHGs (4.9) were perceived more comfortable than both PG-3 (3.2, p < 0.001) and PG-5 (2.9, p < 0.001).

**Fig 2:**
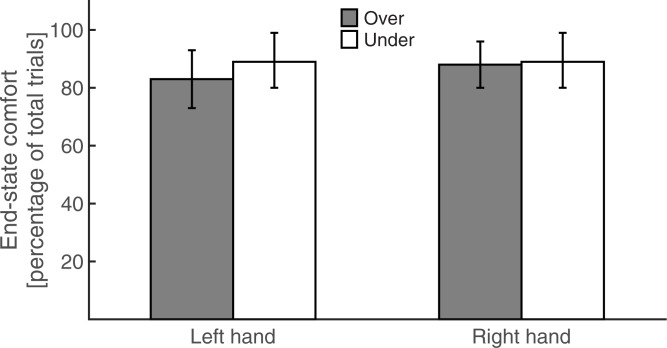
Percentage of trials in which participants satisfied end-state comfort as a function of hand and movement path during unimanual conditions. Error bars represent 95% confidence intervals.

Participants selected their grips to satisfy end-state comfort on the majority of the unimanual trials (Left hand over: 83%, Right hand over: 88%, Left hand under: 89%, Right hand under: 89%, Fig 2). The Friedman test showed that end-state comfort satisfaction did not differ significantly across the unimanual conditions, χ^2^(3) = 7.00, p = 0.072.

**Fig 3:**
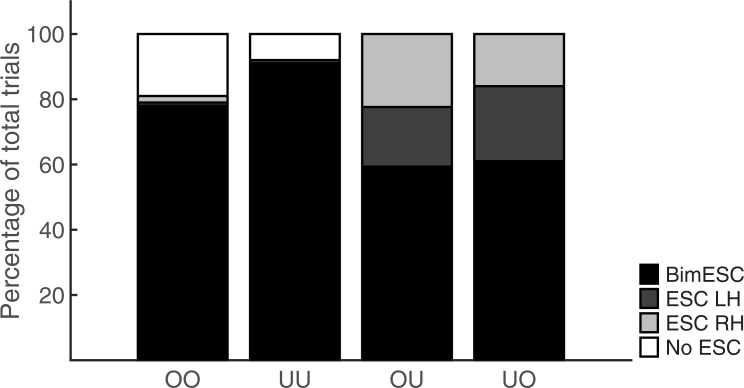
Percentage of trials in which participants satisfied bimanual end-state comfort (Bim ESC), end-state comfort for the left hand only (ESC LH), end-state comfort for the right hand only (ESC RH), or for neither hand (No ESC) during bimanual conditions.

Fig 3 shows the proportion of trials in which participants satisfied bimanual end-state comfort (BIM ESC), end-state comfort for the left hand only (ESH LH), end-state comfort for the right-hand only (ESC RH), or for neither hand (No ESC) during bimanual trials. To contrast the End-state dominance hypothesis and the Means dominance hypothesis, we compared the proportion of trials in which bimanual end-state comfort was satisfied for each of the four bimanual conditions (OO, UU, OU, UO). Participants adopted initial grips that resulted in bimanual end-state comfort in 78%, 91%, 59%, and 61% for the conditions OO, UU, OU, and UO, respectively. A Friedman test showed significant differences in bimanual end-state comfort across the four conditions, χ^2^(3) = 39.889, p < 0.001. Pairwise comparisons with adjusted p-values (Dunn-Bonferroni corrected) showed that bimanual end-state comfort was significantly higher for the UU condition than for the OU condition (Z = 4.665, p < 0.001, r = 0.76) and for the UO condition (Z = 3.510, p = 0.003, r = 0.57). Bimanual end-state comfort was also higher for the OO condition compared to the OU condition (Z = 3.065, p = 0.013, r = 0.50). The difference between OO and UO was not significant (Z = 1.910, p = 0.337, r = 0.31). Furthermore, neither the two congruent conditions (OO vs. UU, Z = - 1.599, p = 0.658, r = 0.26) nor the two incongruent conditions (OU vs. UO, Z = -1.155, p = 1.000, r = 0.19) differed significantly from each other. In sum, bimanual end-state comfort was somewhat reduced in the incongruent conditions compared to the congruent conditions.

### Reaction time (RT) and movement time (MT)

RTs and MTs were analyzed using separate 3×2×2 repeated measures ANOVAs with the within-subject factors task (unimanual, bimanual congruent, bimanual incongruent), hand (left, right), and movement path (over, under). If the sphericity assumption was violated, the Greenhouse-Geisser correction was applied, but uncorrected degrees of freedom are reported.

**Fig 4:**
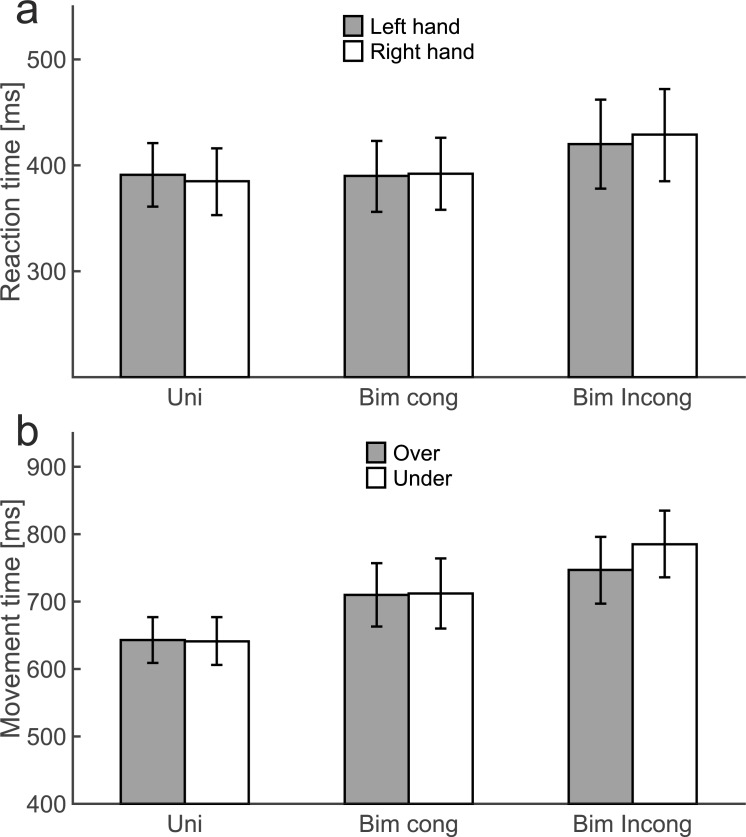
Mean RTs as a function of task and hand (A) and mean MTs as a function of task and path (B). Error bars represent 95% confidence intervals.

For RT, there was a main effect of task, *F*(2,74) = 5.44, *p* = 0.010, *f* = 0.38. Post hoc pair wise comparisons (Bonferroni corrected) revealed that RTs were slower for bimanual incongruent trials (424 ms) compared to bimanual congruent trials (391 ms, p = 0.004) and unimanual trials (388 ms, p = 0.050). In addition, the task × hand interaction was significant, *F*(2,74) = 5.49, *p* = 0.019, *f* = 0.38 (Fig 4A). Simple effect analyses showed RTs were faster for the left hand (420 ms) than the right hand (429 ms) during bimanual incongruent conditions (*p* = 0.005), but not during unimanual or bimanual congruent conditions (both *p* > 0.3).

For MT, the main effect of task was also significant, *F*(2,74) = 52.54, *p* < 0.001, *f* = 1.19. MTs were 642 ms, 711 ms, and 766 ms for unimanual, bimanual congruent, and bimanual incongruent conditions, respectively. Pairwise comparions (Bonferroni correct) indicated that all conditions differed significantly from each other (all *p* < 0.001). Furthermore, the main effect of path and the task × path interaction were significant [*F*(1,37) = 7.86, *p* = 0.008, *f* = 0.46; *F*(2,74) = 12.67, *p* < 0.001, *f* = 0.59, Fig 4B]. Simple effect analyses revealed faster MTs when the object(s) had to be moved over (747 ms) compared to under the obstacle (785 ms) during bimanual incongruent trials (*p* < 0.001), but not during bimanual congruent or unimanual trials (both *p* > 0.6). No other main effects or interactions were significant.

In sum, both RTs and MTs were significantly slower for bimanual incongruent compared to bimanual congruent conditions (and unimanual conditions), indicative of intermanual interference.

## Discussion

The present study examined whether goal-related planning constraints (i.e., bimanual end-state comfort) take precedence over means-related processes (i.e., bimanual symmetry) if this requires anticipating different final postures for the two hands. Participants performed a bimanual object manipulation task, where they simultaneously reached and grasped two objects from two start locations and placed them at two target locations, by transporting them either over or under an obstacle. When the transport paths for the two objects were congruent, we found that participants satisfied bimanual end-state comfort (and bimanual symmetry) on the majority of trials. That is, participants predominantly adopted a PG for either hand, when the objects were transported over the obstacle, and a WHG for either hand, when the objects were transported under the obstacle. In contrast, when the transport paths for the two objects differed, we found that the tendency to satisfy bimanual end-state comfort was reduced. Furthermore, both RTs and MTs were significantly slower for bimanual incongruent compared to bimanual congruent conditions.

Taken together, the grip selection data in conjunction with the results from the RT and MT analyses seem to be at odds with both of our original hypotheses (i.e., End-state dominance and Means dominance).

First, the Means dominance hypothesis would predict no bimanual end-state comfort during bimanual incongruent conditions. Furthermore, although the RT and MT data are indicative of intermanual interference, these data are difficult to interpret, because the reach pathways for the two hands differed in the bimanual incongruent conditions. Thus, it is equally possible that the differences in RT and MT are (at least partly) due to planning and executing two different reach pathways rather than planning and executing two different grip postures. In addition, the design of the experiment (the low number of trials per condition) also demands that the RT/MT data should be treated with caution.

Second, the data also seem to be incompatible with the End-state dominance hypothesis, as this hypothesis would predict similarly high bimanual end-state comfort during bimanual incongruent and congruent conditions. However, this conclusion might be premature given that participants did not exhibit fully-fledged end-state comfort satisfaction during unimanual trials. Hence, the differences in end-state comfort values between the bimanual congruent and incongruent conditions might be traced back to inherent differences in end-state comfort sensitivity across task conditions (i.e., hand and/or movement path) within and between participants. For example, consider the extreme case that one participant is satisfying ESC in 100% of the unimanual trials when moving the object over the obstacle, but in 0% of the unimanual trials when moving the object under the obstacle, whereas a second participant exhibits the exact opposite pattern (i.e., 0% ESC for over and 100% for under). If movements for each hand can be planned independently, one would predict the first participant to show 100% Bim ESC for the OO condition and 0% Bim ESC for the other bimanual conditions, and the second participant to show 100% Bim ESC for the UU condition and 0% Bim ESC for the other bimanual conditions. Averaging across these two participants would then lead to 50% Bim ESC for both bimanual congruent conditions (i.e., OO and UU) and 0% Bim ESC for both bimanual incongruent conditions (i.e., OU and UO). Consequently, in this scenario the End-state dominance hypothesis would also predict differences in ESC between the bimanual congruent and incongruent conditions. Third, it is also possible that the data represent a tradeoff between goal-related and means-related planning processes.

At first glance, this latter explanation appears to be supported by most of the previous studies examining grip posture planning during bimanual object manipulation tasks (but see [17] for support of the End-state dominance hypothesis). In these studies, participants typically grasped the objects exclusively with WHGs and potential intermanual inference was assessed by manipulating the required object end orientation (see [28] for a review). A converging result of these tasks was that participants typically selected initial grips that satisfied end-state comfort for both hands (regardless of whether bimanual end-state comfort coincided with bimanual symmetry) for as long as the required object end-orientations were identical [14,16,17,29–31]. In contrast, when the required object end orientations differed, these studies yielded mixed results. In these instances, the tendency to satisfy bimanual end-state comfort depended on the rotation requirements. Specifically, when the required degree of object rotation was identical, participants selected grips that resulted in bimanual end-state comfort [16,17]. However, when different degrees of object rotations were required, the tendency to satisfy end-state comfort for both hands decreased considerably [32], sometimes even to chance level [14,29], thus suggesting that both goal-directed (i.e., bimanual end-state comfort) and means-directed (i.e. movement symmetry) constraints influence grip posture planning.

However, as these studies either did not assess baseline performance using unimanual trials [14,16,29,31–33] or did not explicitly relate unimanual performance to bimanual performance [15,30,34], it is still possible that these results can also be explained equally well with the End-state dominance hypothesis.

### Model of bimanual grip selection

To provide additional insights into the mechanisms underlying grip selection during bimanual object manipulation tasks, we created a simple formal model (see S1 Appendix for Model specification) that uses the performance during unimanual conditions (i.e., unimanual ESC) to predict the performance during the bimanual conditions (i.e., Bim ESC, ESC LH, ESC RH, No ESC). Please note that we observed a strong correlation between ESC satisfaction in unimanual trials and Bim ESC (r = 0.70, p < 0.001), thus lending credit to the assumption that bimanual performance can be predicted by unimanual performance.

According to the model, grip selection during bimanual object manipulation results from the relative weighing of goal-related and means-related planning processes. Thus, the model was comprised of two additive terms, one representing goal-related influences, and the other representing means-related influences. Each term was assigned a weight factor, ω_Goal_ and ω_Means_, respectively, which lay in the range of 0–1, and summed to 1, constituting one free parameter ω_Planning_ (true of all weights). Furthermore, within the means-related term, we included two more weight factors, ω_LH_ and ω_RH_, that assessed whether one hand’s grip selection would consistently influence the other hand’s grip selection, constituting a second free parameter ω_Hand_.

**Fig 5:**
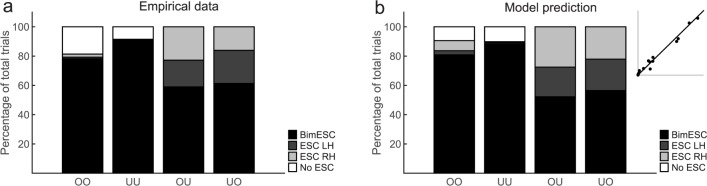
Percentage of trials in which participants satisfied bimanual end-state comfort (Bim ESC), end-state comfort for the left hand only (ESC LH), end-state comfort for the right hand only (ESC RH), or for neither hand (No ESC) during bimanual conditions. (a) Observed data, (b) Model prediction. The inset plots the model output (y-axis) against the empirical data (x-axis). Both axes from the insets range from 0 to 1.

We evaluated the model using cross-validation on the level of the individual participant. To this end, we repeated the following step 100 times: First, we sampled a subset of 50–66% of unimanual and bimanual trials for each participant. Using these training data, we then fitted the values for the two free parameters (ω_Planning_, ω_Hand_) individually for each participant via an iterative search algorithm, using the matlab function lsqnonlin (https://de.mathworks.com/help/optim/ug/lsqnonlin.html) to find the values that minimize the root mean square error for each participant. The parameters obtained from the optimal fitting procedure were then applied to the remaining 33–50% of unimanual trials (unimanual test data) to predict performance during bimanual trials. The model was then validated by comparing the model’s prediction with the performance of the remaining 33–50% of bimanual trials (bimanual test data). As shown in Table 2, the model provides a reasonably good fit (R^2^ = 0.69 and RSME = 20%, averaged over all participants) and resembles the observed data quite well (cp. Fig 5A and 5B).

**Table 2:** Means ± 1 standard deviation (and range) of model fits and parameter estimates.

R^2^	0.69 ± 0.27 (0.06–1.00)
RMSE	20% ± 13% (0–49)
ω_Goal_	0.65 ± 0.30 (0.00–1.00)
ω_Means_	0.35 ± 0.30 (0.00–1.00)
ω_LH_	0.51 ± 0.26 (0.03–1.00)
ω_RH_	0.49 ± 0.26 (0.03–1.00)

Taken together, the data are reasonably well explained by a model that considers bimanual grip posture planning as a tradeoff between end-state planning and means-related planning processes, with participants assigning different weights to each of these to processes. On average, participants assigned more weight to goal-related constraints (0.65) than to means-related constraints (0.35). However, there were considerable differences between participants in the weights assigned from the optimal fitting procedure. Indeed, the range of ω_Independent_ was from 0–1 (see Table 2), indicating that some participants apparently put more emphasis on end-state comfort, whereas others assigned more weight to the adoption of identical grips.

Hence, our results indicate that goal-directed planning constraints (i.e., bimanual end-state comfort) will not strictly take precedence over means-related constraints (i.e., bimanual symmetry) if this requires anticipating different final grips. As such, the present findings not only complement previous studies, but also provide more conclusive evidence for their interpretations, because by predicting performance in bimanual conditions using an unimanual baseline performance measurement, inherent differences in end-state comfort sensitivity across task conditions within and between participants, that might cause differences in grip selection between bimanual congruent and bimanual incongruent conditions, are explicitly taken into account.

Our data accord with previous proposals emphasizing that bimanual grip posture planning relies on a flexible constraint hierarchy and that different constraints can take on different degrees of importance depending on external (e.g., nature of the task) and internal (e.g., experience) influences [15,35,36]. Consequently, one might speculate that bimanual end-state comfort *per se* is not sufficient to provide a predominant constraint in action selection. Rather, it seems important to generate common final grips [17] and/or similar object end-orientations [24]. Thus, action goals may not be represented in terms of bimanual end-state comfort as an abstract concept, but rather in terms of final goal orientations and/or grips. This notion is in line with the posture-based planning account [3]. According to this model, action selection involves assigning weights to constraints, from most to least important, depending on what needs to be achieved. That way, the weighting of the constraints defines the task to be performed, as represented by the actor. In the context of reaching and grasping, these constraints pertain to features of body positions during forthcoming movements and particularly to goal postures that can be adopted when the movement ends. These goal postures are planned before the movements are planned, by evaluating recently adopted goal postures with respect to the constraint hierarchy (i.e., the ones that satisfies most constraints). If there is sufficient time, a potentially better goal posture is sought. In the present task, one might speculate that it was of primary importance to adopt postures that allow for transporting the object from the start to the target positions without colliding with the obstacle, whereas the adoption of comfortable end-states was assigned a lower weight, and this constraint was more likely to be satisfied with increasing time. In line with this speculation, we observed a significant correlation between differences in BimESC satisfaction (incongruent–congruent conditions) and RT differences between these conditions (r = 0.43, p = 0.008), meaning that a smaller decrease in BimESC during incongruent compared to congruent conditions was associated with larger RT cost.

In sum, the results of the present study indicate that goal-related planning constraints (i.e., bimanual end-state comfort) do not strictly take precedence over means-related constraints (i.e., bimanual symmetry) if this requires anticipating different final grips. Thus, bimanual end-state comfort *per se* does not provide a predominant constraint in action selection, by which sensorimotor interference can be reduced. In line with the proposal that bimanual grip planning relies on a flexible constraint hierarchy, a simple formal model that considers bimanual grip posture planning as a tradeoff between goal-related and means-related planning processes can explain our results reasonably well.

## Supporting information

S1 Appendix Model specification. (DOCX)

S1 File Raw data. (TXT)

## References

[1] Rosenbaum DA. Human motor control 2nd ed Amsterdam: Elsevier; 2010.

[2] Rosenbaum DA, Chapman KM, Coelho CJ, Gong L, Studenka BE. Choosing Actions. Front. Psychol. 2013; 4 doi: 10.3389/fpsyg.2013.00004 23761769 10.3389/fpsyg.2013.00273 PMC3669743

[3] Rosenbaum DA, Meulenbroek R, Vaughan J, Jansen C. Posture-based motion planning: applications to grasping. Psychological Review. 2001; 108: 709–734. 11699114 10.1037/0033-295x.108.4.709

[4] Rosenbaum DA, Chapman KM, Weigelt M, Weiss DJ, van der Wel R. Cognition, Action, and Object Manipulation. Psychological Bulletin. 2012; 138: 924–946. doi: 10.1037/a0027839 22448912 10.1037/a0027839 PMC3389205

[5] Rosenbaum DA, Marchak F, Barnes HJ, Vaughan J, Slotta JD, Jorgensen MJ. Constraints for action selection: overhand versus underhand grips In: Jeannerod M, editor. Attention and performance XIII. Hillsdale: Erlbaum; 1990 pp. 321–342.

[6] Cohen RG, Rosenbaum DA. Where grasps are made reveals how grasps are planned: generation and recall of motor plans. Experimental Brain Research. 2004; 157: 486–495. doi: 10.1007/s00221-004-1862-9 15071711 10.1007/s00221-004-1862-9

[7] Herbort O, Butz MV. Planning and control of hand orientation in grasping movements. Experimental Brain Research. 2010; 202: 867–878. doi: 10.1007/s00221-010-2191-9 20195848 10.1007/s00221-010-2191-9

[8] Hughes CML, Seegelke C, Spiegel MA, Oehmichen C, Hammes J, Schack T. Corrections in grasp posture in response to modifications of action goals. PLOS ONE. 2012; 7: e43015 doi: 10.1371/journal.pone.0043015 22970119 10.1371/journal.pone.0043015 PMC3435384

[9] Seegelke C, Hughes CML, Schack T. An investigation into manual asymmetries in grasp behavior and kinematics during an object manipulation task. Experimental Brain Research. 2011; 215: 65–75. doi: 10.1007/s00221-011-2872-z 21938544 10.1007/s00221-011-2872-z

[10] Seegelke C, Hughes CML, Schütz C, Schack T. Individual differences in motor planning during a multi-segment object manipulation task. Experimental Brain Research. 2012; 222: 125–136. doi: 10.1007/s00221-012-3203-8 22885998 10.1007/s00221-012-3203-8

[11] Seegelke C, Hughes CM, Knoblauch A, Schack T. Grasp posture planning during multi-segment object manipulation tasks—Interaction between cognitive and biomechanical factors. Acta Psychologica. 2013; 144: 513–521. doi: 10.1016/j.actpsy.2013.09.002 24095853 10.1016/j.actpsy.2013.09.002

[12] Coelho CJ, Studenka BE, Rosenbaum DA. End-state comfort trumps handedness in object manipulation. Journal of Experimental Psychology: Human Perception and Performance. 2014; 40: 718–730. doi: 10.1037/a0034990 24294873 10.1037/a0034990

[13] Chapman KM, Rosenbaum DA. Bimanual comfort depends on how extreme either hand's posture is, not on which hand is in the more extreme posture. Psychological Research. 2017; 81: 332–341. doi: 10.1007/s00426-015-0708-3 26409467 10.1007/s00426-015-0708-3

[14] Hughes CM, Seegelke C, Reißig P. Problems in planning bimanually incongruent grasp postures relate to simultaneous response specification processes. Brain and Cognition. 2014; 87: 22–29. doi: 10.1016/j.bandc.2014.02.010 24650762 10.1016/j.bandc.2014.02.010

[15] Hughes CML, Franz EA. Goal-related planning constraints in bimanual grasping and placing of objects. Experimental Brain Research. 2008; 188: 541–550. doi: 10.1007/s00221-008-1387-8 18443769 10.1007/s00221-008-1387-8

[16] Hughes CML, Seegelke C. Perturbations in Action Goal Influence Bimanual Grasp Posture Planning. Journal of Motor Behavior. 2013; 45: 473–478. doi: 10.1080/00222895.2013.828677 24006878 10.1080/00222895.2013.828677

[17] Weigelt M, Kunde W, Prinz W. End-state comfort in bimanual object manipulation. Experimental Psychology (formerly Zeitschrift für Experimentelle Psychologie). 2006; 53: 143–148. doi: 10.1027/1618-3169.53.2.143 16909939 10.1027/1618-3169.53.2.143

[18] Franz EA, Zelaznik H, McCabe G. Spatial topological constraints in a bimanual task. Acta Psychologica. 1991; 77: 137–151. 1759589 10.1016/0001-6918(91)90028-x

[19] Kelso J, Southard D, Goodman D. On the nature of human interlimb coordination. Science. 1979; 203: 1029–1031. 424729 10.1126/science.424729

[20] Heuer H. Structural constraints on bimanual movements. Psychol Res. 1993; 55: 83–98. 8356202 10.1007/BF00419639

[21] Spijkers W, Heuer H, Kleinsorge T, van der Loo Hanno. Preparation of bimanual movements with same and different amplitudes: specification interference as revealed by reaction time. Acta Psychol (Amst). 1997; 96: 207–227.

[22] Oliveira F, Ivry R. The representation of action: insights from bimanual coordination. Current Directions in Psychological Science. 2008; 17: 130–135. doi: 10.1111/j.1467-8721.2008.00562.x 19606276 10.1111/j.1467-8721.2008.00562.x PMC2709871

[23] Wenderoth N, Weigelt M. Visual cues influence motor coordination: behavioral results and potential neural mechanisms mediating perception–action coupling and response selection. Progress in Brain Research: 179–188. Accessed 18 September 2012. 10.1016/S0079-6123(09)01315-6 19477339

[24] Kunde W, Weigelt M. Goal Congruency in Bimanual Object Manipulation. Journal of Experimental Psychology: Human Perception and Performance. 2005; 31: 145–156. doi: 10.1037/0096-1523.31.1.145 15709869 10.1037/0096-1523.31.1.145

[25] Napier J. The prehensile movements of the human hand. The Journal of Bone and Joint Surgery. 1956; 38 B: 902–913. 13376678 10.1302/0301-620X.38B4.902

[26] Dragovic M. Towards an improved measure of the Edinburgh Handedness Inventory. A one-factor congeneric measurement model using confirmatory factor analysis. Laterality. 2004; 9: 411–419. doi: 10.1080/13576500342000248 15513238 10.1080/13576500342000248

[27] Woltring HJ. A Fortran package for generalized, crossvalidatory spline smoothing and differentiation. Advances in Engineering Software. 1986; 8: 104–133.

[28] Seegelke C, Hughes CML, Schack T. Manual (a)symmetries in grasp posture planning: a short review. Front. Psychol. 2014; 5 doi: 10.3389/fpsyg.2014.00005 25566153 10.3389/fpsyg.2014.01480 PMC4265983

[29] Hughes CM, Reißig P, Seegelke C. Motor planning and execution in left- and right-handed individuals during a bimanual grasping and placing task. Acta Psychologica. 2011; 138: 111–118. doi: 10.1016/j.actpsy.2011.05.013 21663882 10.1016/j.actpsy.2011.05.013

[30] Hughes CML, Haddad JM, Franz EA, Zelaznik HN, Ryu JH. Physically coupling two objects in a bimanual task alters kinematics but not end-state comfort. Experimental Brain Research. 2011; 211: 219–229. doi: 10.1007/s00221-011-2673-4 21484393 10.1007/s00221-011-2673-4

[31] Hughes CML, Seegelke C, Reißig P, Schütz C. Effects of stimulus cueing on bimanual grasp posture planning. Experimental Brain Research. 2012; 219: 391–401. doi: 10.1007/s00221-012-3100-1 22562588 10.1007/s00221-012-3100-1

[32] Janssen L, Crajé C, Weigelt M, Steenbergen B. Motor planning in bimanual object manipulation: two plans for two hands. Motor Control. 2010; 14: 240–254. 20484772 10.1123/mcj.14.2.240

[33] Janssen L, Meulenbroek RGJ, Steenbergen B. Behavioral evidence for left-hemisphere specialization of motor planning. Experimental Brain Research. 2011; 209: 65–72. doi: 10.1007/s00221-010-2519-5 21184219 10.1007/s00221-010-2519-5 PMC3035772

[34] Janssen L, Beuting M, Meulenbroek R, Steenbergen B. Combined effects of planning and execution constraints on bimanual task performance. Experimental Brain Research. 2009; 192: 61–73. doi: 10.1007/s00221-008-1554-y 18751972 10.1007/s00221-008-1554-y

[35] Hughes CM, Seegelke C, Schack T. The influence of initial and final precision on motor planning: individual differences in end-state comfort during unimanual grasping and placing. Journal of Motor Behavior. 2012; 44: 195–201. doi: 10.1080/00222895.2012.672483 22551086 10.1080/00222895.2012.672483

[36] van der Wel R.P.R.D, Rosenbaum DA. Bimanual grasp planning reflects changing rather than fixed constraint dominance. Experimental Brain Research. 2010; 205: 351–362. doi: 10.1007/s00221-010-2368-2 20658129 10.1007/s00221-010-2368-2 PMC2923322

