# Supplementary material for: Anticipating different grips reduces bimanual end-state comfort: A tradeoff between goal-related and means-related planning processes
Source: PLoS One. 2018 Jan 8;13(1):e0190586. doi: 10.1371/journal.pone.0190586 (PMC5757994; doi:10.1371/journal.pone.0190586)
Supplement: S1 Appendix — (DOCX) [file pone.0190586.s001.docx]

**S1 Appendix. Model specification.** The model was comprised of two additive terms for each of the four bimanual conditions (i.e., OO, UU, OU, UO) and each of the four possible outcomes of each condition: Bimanual end-state comfort (Bim ESC), end-state comfort for the left hand only (ESC LH), end-state comfort for the right hand only (ESC RH), and end-state comfort for neither hand (No ESC). The first term described independent planning and the second term described intermanual inference, and each term was assigned a weight factor, ω_Goal_ and ω_Means_, respectively. Furthermore, within the interference term, we included two more weight factors, ω_LH_ and ω_RH_, that assessed whether one hand’s grip selection would consistently influence the other hand’s grip selection. The inputs to the model were the proportions of trials in which end-state comfort was satisfied during the four unimanual conditions.

**Bimanual end-state comfort.** For the bimanual congruent conditions (OO, UU), Bim ESC was computed as:

${BimESC}_{L,R}=\omega_{Goal}\left( {ESC}_{Uni_{L}}{ESC}_{Uni_{R}} \right)+\omega_{Means}(\omega_{LH}{ESC}_{Uni_{L}}+\omega_{RH}{ESC}_{Uni_{R}})$

where L is the movement path for the left hand and R is the movement path for the right hand.

For the bimanual incongruent conditions (OU, UO), the interference term is removed as the Intermanual interference hypothesis would predict no bimanual end-state comfort.

$${BimESC}_{L,R}=\omega_{Goal}({ESC}_{Uni_{L}}{ESC}_{Uni_{R}})$$

**ESC for the left/right hand only.** For the bimanual congruent conditions (OO, UU), the interference term is removed as the Intermanual interference hypothesis would predict no bimanual end-state comfort.

$${ESC LH}_{L,R}=\omega_{Goal}[{ESC}_{Uni_{L}}\left( 1-{ESC}_{Uni_{R}} \right)]$$

$${ESC RH}_{L,R}=\omega_{Goal}[\left( 1-{ESC}_{Uni_{L}} \right){ESC}_{Uni_{R}}]$$

For the bimanual incongruent conditions (OU, UO), ESC LH and ESC RH was computed as:

$${ESC LH}_{L,R}=\omega_{Goal}\left[ {ESC}_{Uni_{L}}\left( 1-{ESC}_{Uni_{R}} \right) \right]+\omega_{Means}[\omega_{LH}{ESC}_{Uni_{L}}+\omega_{RH}\left( 1-{ESC}_{Uni_{R}} \right)]$$

$${ESC RH}_{L,R}=\omega_{Goal}\left[ \left( 1-{ESC}_{Uni_{L}} \right){ESC}_{Uni_{R}} \right]+\omega_{Means}[\omega_{LH}\left( 1-{ESC}_{Uni_{L}} \right)+\omega_{RH}{ESC}_{Uni_{R}}]$$

**End-state comfort for neither hand.** For the bimanual congruent conditions (OO, UU), No ESC is computed using the proportion of unimanual trials in which end-state comfort was not achieved (i.e., 1 – ESC_Uni_):

$${No ESC}_{L,R}=\omega_{Goal}\left[ \left( 1-{ESC}_{Uni_{L}} \right)\left( 1-{ESC}_{Uni_{R}} \right) \right]+\omega_{Means}[\omega_{LH}\left( 1-{ESC}_{Uni_{L}} \right)+\omega_{RH}\left( 1-{ESC}_{Uni_{R}} \right)]$$

For the bimanual incongruent conditions (OU, UO), the interference term is removed as the Intermanual interference hypothesis would always predict end-state comfort for one hand

$${No ESC}_{L,R}=\omega_{Goal}[\left( 1-{ESC}_{Uni_{L}} \right)\left( 1-{ESC}_{Uni_{R}} \right)]$$
